# Augmented Osteogenic Responses in Human Aortic Valve Cells Exposed to oxLDL and TLR4 Agonist: A Mechanistic Role of Notch1 and NF-κB Interaction

**DOI:** 10.1371/journal.pone.0095400

**Published:** 2014-05-08

**Authors:** Qingchun Zeng, Rui Song, Lihua Ao, Dingli Xu, Neil Venardos, David A. Fullerton, Xianzhong Meng

**Affiliations:** 1 Department of Surgery, University of Colorado Denver, Aurora, Colorado, United States of America; 2 Department of Cardiology, Nanfang Hospital, Southern Medical University, Guangzhou, Guangdong, China; 3 Department of Pathophysiology, Southern Medical University, Guangzhou, Guangdong, China; University of Cincinnati, College of Medicine, United States of America

## Abstract

Aortic valve calcification causes the progression of calcific aortic valve disease (CAVD). Stimulation of aortic valve interstitial cells (AVICs) with lipopolysaccharide (LPS) up-regulates the expression of osteogenic mediators, and NF-κB plays a central role in mediating AVIC osteogenic responses to Toll-like receptor 4 (TLR4) stimulation. Diseased aortic valves exhibit greater levels of oxidized low-density lipoprotein (oxLDL). This study tested the hypothesis that oxLDL augments the osteogenic responses in human AVICs through modulation of NF-κB and Notch1 activation. AVICs isolated from normal human aortic valves were treated with LPS (0.1 µg/ml), oxLDL (20 µg/ml) or LPS plus oxLDL for 48 h. OxLDL alone increased cellular bone morphogenetic protein-2 (BMP-2) levels while it had no effect on alkaline phosphatase (ALP) levels. Cells exposed to LPS plus oxLDL produced higher levels of BMP-2 and ALP than cells exposed to LPS alone. Further, LPS plus oxLDL induced greater NF-κB activation, and inhibition of NF-κB markedly reduced the expression of BMP-2 and ALP in cells treated with LPS plus oxLDL. OxLDL also induced Notch1 activation and resulted in augmented Notch1 activation when it was combined with LPS. Inhibition of Notch1 cleavage attenuated NF-κB activation induced by LPS plus oxLDL, and inhibition of NF-κB suppressed the expression of BMP-2 and ALP induced by the synergistic effect of Jagged1 and LPS. These findings demonstrate that oxLDL up-regulates BMP-2 expression in human AVICs and synergizes with LPS to elicit augmented AVIC osteogenic responses. OxLDL exerts its effect through modulation of the Notch1-NF-κB signaling cascade. Thus, oxLDL may play a role in the mechanism underlying CAVD progression.

## Introduction

Calcific aortic valve disease (CAVD) is one of the leading cardiovascular diseases in the United States [1], [2]. Despite the prevalence of this disease, the mechanisms underlying its development and progression are not well understood. In particular, the cellular and molecular mechanisms by which the aortic valve leaflets become calcified and stenotic are unclear. A thorough understanding of these mechanisms will be required to identify potential targets for pharmacological limitation of CAVD progression.

Aortic valve interstitial cells (AVICs) are the principal cells in aortic valve leaflet, and the inflammatory and osteogenic responses of AVICs have been implicated in CAVD development and/or progression [3]. We found that stimulation of Toll-like receptor 4 (TLR4) in human AVICs with lipopolysaccharide (LPS) not only induces the production of inflammatory mediators, but also up-regulates the expression of osteogenic factors, including bone morphogenetic protein-2 (BMP-2) and alkaline phosphatase (ALP) [4], [5]. This finding uncovered a novel role for TLR4 in the regulation of the osteogenic responses in AVICs. Several studies found that bacterial agents associated with chronic periodontal infection are present in diseased aortic valves [6], [7], [8], [9]. Furthermore, inoculation of rabbits with oral bacteria was shown to cause aortic valve lesions [10]. Investigation of the mechanisms underlying AVIC osteogenic responses to pro-inflammatory stimuli could provide insights into the mechanisms of CAVD pathogenesis and progression.

Oxidized low-density lipoprotein (oxLDL) accumulation in the vascular wall provokes atherosclerotic calcification [11]. Increased levels of oxLDL in blood have been reported to correlate with aortic valve fibrosis and calcification [12]. A number of studies found oxLDL accumulation in stenotic aortic valves [13], [14], [15], [16]. In addition, we observed that oxLDL up-regulates BMP-2 expression in human coronary artery endothelial cells [17]. Currently, it remains unclear whether oxLDL plays a role in the osteogenic responses in aortic valve cells. Interestingly, several studies indicate that oxLDL modulates TLR4 expression or signaling in macrophages, resulting in an enhanced cytokine response to TLR4 stimulation [18], [19], [20], [21], [22]. While oxLDL and bacterial products may co-exist in diseased aortic valves, the effect of oxLDL on TLR4-mediated osteogenic responses in human AVICs has not been determined. We hypothesized that oxLDL synergizes with TLR4 agonist to induce augmented osteogenic responses in human AVICs.

Notch receptors undergo proteolytic cleavage upon ligand binding, leading to the release of intracellular domains (NICDs) that control cell fate and modulate cell functions [23]. Bacterial lipopeptide and LPS have been found to induce Notch1 activation in macrophages [24]. Inhibition of γ-secretase, which processes Notch1 to release NICD1, reduces LPS-induced expression of pro-inflammatory cytokines in macrophages [25]. Thus, Notch1 signaling plays a role in TLR4-mediated inflammatory response. We recently observed that Notch1 interacts with the TLR4 signaling pathway, specifically the NF-κB pathway and plays an important role in modulating TLR4-mediated inflammatory and osteogenic responses in human AVICs [26], [27]. We further hypothesized that the synergy between oxLDL and LPS involves the enhancement of Notch1 activation.

This hypothesis-driven study determined: 1) the effect of oxLDL on BMP-2 and ALP expression in human AVICs stimulated with TLR4 agonist LPS, 2) the impact of oxLDL on Notch1 and NF-κB activation, and 3) the role of Notch1 in mediating the effect of oxLDL on NF-κB activation and the expression of BMP-2 and ALP.

## Materials and Methods

This study was approved by the Institutional Review Board of University of Colorado Denver. All participants provided written consent to participate in this study.

### Materials

Notch1 siRNA, scrambled siRNA and transfection reagents were purchased from Santa Cruz Biotechnology, Inc. (Santa Cruz, CA). The antibody against bone ALP was purchased from Abcam (San Francisco, CA). Antibodies against Notch1, NICD1, β-actin, phosphorylated NF-κB p65 (Ser536) and total NF-κB p65 were purchased from Cell Signaling, Inc. (Beverly, MA). Medium 199 was purchased from Lonza (Walkersville, MD). Recombinant Jagged1 and cytokine ELISA kits were purchased from R&D System (Minneapolis, MN). Jagged1 ELISA kit was purchased from Uscn Life Science Inc. (Germany). NF-κB p65 DNA-binding activity assay kit was purchased from Active Motif (Carlsbad, CA). OxLDL was purchased from Biomedical Technologies Inc. (Stoughton, MA). SN50 and BAY11-7082 were purchased from Enzo Life Sciences Inc. (Farmingdale, NY). GSI1 (z-leu-leu-nle-CHO) was purchased from Calbiochem (San Diego, CA). DAPT, LPS (E. coli 0111:B4) and other chemicals and reagents were purchased from Sigma-Aldrich Chemical Co (St Louis, MO).

### Cell Isolation and Culture

Aortic valve leaflets were collected from the explanted hearts of 10 patients (7 males and 3 females, all with cardiomyopathy, mean age 56±10.2 years) undergoing heart transplantation at the University of Colorado Hospital. All valve leaflets were thin and histologically normal. This study was approved by the COMIRB of University of Colorado Denver. All patients gave informed consent for the use of their valves for this study.

AVICs were isolated and cultured using a previously described method [28] with modification [4]. Briefly, valve leaflets were subjected to sequential digestions with collagenase, and cells were collected by centrifugation. Cells were cultured in M199 growth medium containing penicillin G, streptomycin, amphotericin B and 10% fetal bovine serum. Cells from passages 3 to 5 were used for this study. Cells were subcultured on plates and were treated when they reached 80 to 90% confluence.

### Ethnic Statement

This study was approved by the Institutional Review Board of University of Colorado Denver. All participants provided written consent to participate in this study.

### Cell Treatment

Cells were stimulated with LPS (E. coli 0111:B4, 0.1 µg/ml), oxLDL (20 µg/ml), and LPS plus oxLDL for 48 h for analysis of levels of BMP-2 and ALP. Cells were stimulated with LPS, oxLDL and LPS plus oxLDL for 1 h to 8 h for assessment of NF-κB phosphorylation and Notch1 activation.

To determine the role of Notch1 in the osteogenic response, cells were treated with Notch1 siRNA (60 nM), DAPT (50 µmol/l) or GSI1 (2.0 µmol/l) prior to stimulation. SN50 (100 µg/ml) and BAY11-7082 (2.5 µmol/l) were applied prior to stimulation to inhibit NF-κB.

### Gene Knockdown

Notch1 silencing was performed using the method described previously [26], [27], [29]. Human AVICs were cultured in full growth medium until 60% confluent. Then, cells were incubated with a mixture of siRNA specific to human Notch1 (60 nM) and transfection reagent (6.0 µl per ml of medium) in antibiotic-free medium for 48 h. After transfection, cells were stimulated with LPS plus oxLDL. Control cells were pre-treated with scrambled siRNA and transfection reagent.

### Immunoblotting

Immunoblotting was applied to analyze BMP-2, ALP, NICD1, phosphorylated NF-κB p65, total NF-κB p65 and β-actin. Cells were lysed in a sample buffer (100 mM Tris-HCl, pH 6.8, 2% SDS, 0.02% bromophenol blue and 10% glycerol). Protein samples were separated on gradient (4–20%) minigels and transferred onto nitrocellulose membranes (Bio-Rad Laboratories, Hercules, California). The membranes were blocked with 5% non-fat dry milk solution for 1 h at room temperature. The blocked membranes were incubated in a primary antibody against the protein of interest. After washing with TPBS (PBS containing 0.05% Tween 20), the membranes were incubated with a peroxidase-linked secondary antibody specific to the primary antibody. Following further washes, membranes were treated with enhanced chemiluminescence reagent. Then the membrane was exposed on x-ray film. Image J was used to determine band density.

### Cytochemical Analysis of ALP Activity

To analysis of ALP activity, cells were fixed for 10 min with 3.7% formaldehyde at room temperature. After washing with PBS, fixed cells were incubated for 20 min at room temperature with an ALP substrate solution (0.1 mg/ml of naphthol AS-MX phosphate, 0.5% N, N-dimethylformamide, 2.0 mM MgCl2, and 0.6% fast blue BB salt in 0.1 M Tris-HCl, pH8.5). ALP activity stains were examined and photographed with a Nikon Eclipse TS100 microscope (Tokyo, Japan).

### ELISA

Jagged1 levels in culture supernatants were determined using ELISA kits following manufacturer’s instructions. NF-κB DNA-binding activity was analyzed in cell lysates using an ELISA kit as described previously [30], [31].

### Statistical Analysis

Data are presented as mean ± standard error (SE). Statistical analysis was performed using a StatView software (Abacus Concepts, Calabasas, CA). ANOVA with post hoc Fisher test was performed to analyze differences between experimental groups, and differences were confirmed with Mann-Whitney U tests. Statistical significance was defined as P<0.05.

## Results

### Human AVICs Exhibit Augmented Osteogenic Responses when Exposed to LPS and oxLDL

**Figure 1:**
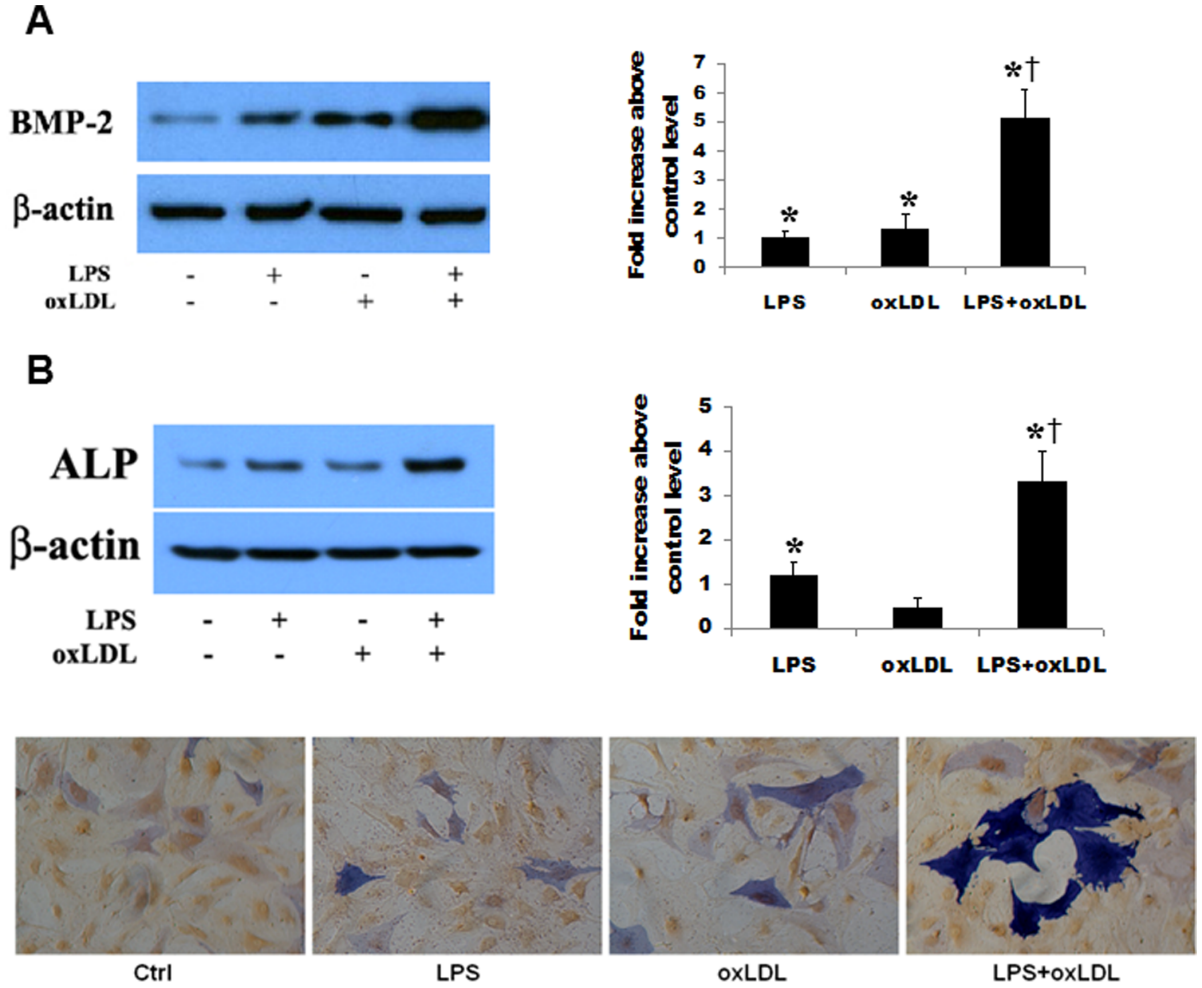
OxLDL and LPS synergize in the induction of the osteogenic responses in human AVICs. Human AVICs were treated with LPS (0.10 µg/ml), oxLDL (20 µg/ml) or LPS plus oxLDL for 48 h. **A**. Representative immunoblots and densitometric data show that oxLDL up-regulates BMP-2 expression. Protein levels of BMP-2 are much higher in cells treated with LPS plus oxLDL than those in cells treated with LPS alone or oxLDL alone. **B**. OxLDL alone has a minimal effect on ALP levels, but cells treated with LPS plus oxLDL produce higher levels of ALP than cells treated with LPS alone. Similarly, ALP activity is higher in cells treated with LPS plus oxLDL. n = 4 separated cell isolates in each group, *P<0.05 vs. untreated control; †*P*<0.05 vs. cells treated with LPS alone or oxLDL alone.

Following stimulation of human AVICs for 48 h with TLR4 agonist LPS (0.1 µg/ml), oxLDL (20 µg/ml) and LPS plus oxLDL, we analyzed the levels of BMP-2 and ALP in cell lysates. For clarity, we presented the results as fold changes above control levels. OxLDL alone increased cellular BMP-2 level by 1.3 folds over the control level (Figure 1A). LPS at the concentration applied induced increases in BMP-2 (1.1 folds over the control level, Figure 1A) and ALP (1.2 folds over the control level, Figure 1B). Interestingly, cells exposed to LPS plus oxLDL produced markedly higher levels of BMP-2 (5.1 folds over the control level, Figure 1A) and ALP (3.3 folds over the control level, Figure 1B). Noticeably, the effect of LPS plus oxLDL on BMP-2 and ALP levels is much greater than the sum of oxLDL and LPS. Similarly, LPS plus oxLDL induced a greater increase in ALP activity than LPS alone (Figure 1B).

### Inhibition of NF-κB Markedly Suppresses the Osteogenic Responses

**Figure 2:**
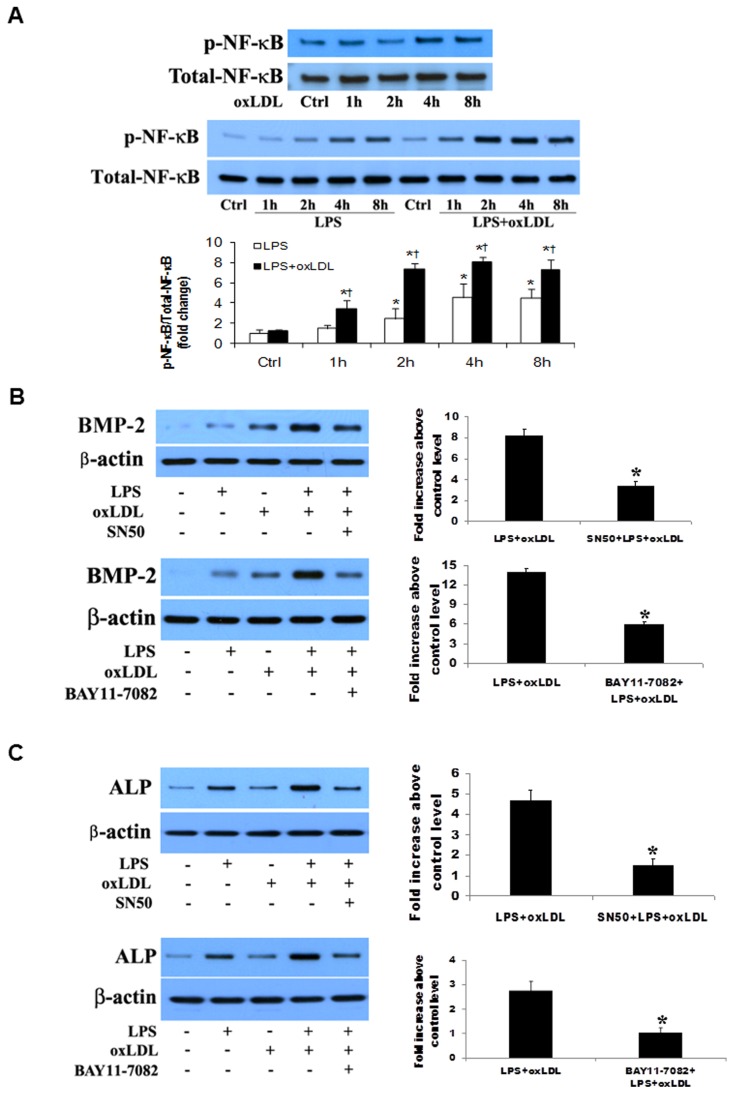
NF-κB plays an important role in the augmented osteogenic responses. **A**. Human AVICs were treated with LPS (0.10 µg/ml), oxLDL (20 µg/ml) or LPS plus oxLDL for 1–8 h. Representative immunoblots and densitometric data (n = 4) show that oxLDL and LPS each induces NF-κB phosphorylation at 4 and 8 h. NF-κB phosphorylation is enhanced when oxLDL and LPS are combined. *P<0.05 vs. untreated control; †*P*<0.05 vs. cells treated with LPS alone. **B** and **C**. Human AVICs were treated with LPS plus oxLDL for 48 h in the absence or presence of NF-κB inhibitor SN50 or BAY11-7082. Representative immunoblots and densitometric data (n = 4) show that inhibition of NF-κB with either of the inhibitors markedly reduces levels of BMP-2 (B) and ALP (C) in cells treated with LPS plus oxLDL. *P<0.05 vs. cells treated with LPS plus oxLDL.

As shown in Figure 2A, NF-κB p65 phosphorylation occurred mainly at 4 and 8 h of stimulation with oxLDL or LPS. NF-κB p65 phosphorylation at 4 and 8 h was greater in cells treated with LPS plus oxLDL. In addition, treatment with LPS plus oxLDL induced NF-κB p65 phosphorylation at 1 and 2 h, resulting in a longer time course of NF-κB p65 phosphorylation. To examine whether inhibition of NF-κB has an effect on the augmented osteogenic responses, we applied specific NF-κB inhibitors, including the cell permeable inhibitory peptide SN50 and IKK inhibitor BAY11-7082, before treatment of cells with LPS plus oxLDL. The results in Figures 2B and 2C show that inhibition of NF-κB with SN50 or BAY11-7082 resulted in a greater than 50% reduction in either BMP-2 (58.8 and 61.8% reduction) or ALP (68.6 and 57.7% reduction). Therefore, the NF-κB pathway plays an important role in the augmented osteogenic responses induced by LPS plus oxLDL.

### Inhibition of Notch1 Attenuates the Osteogenic Responses Induced by LPS plus oxLDL

**Figure 3:**
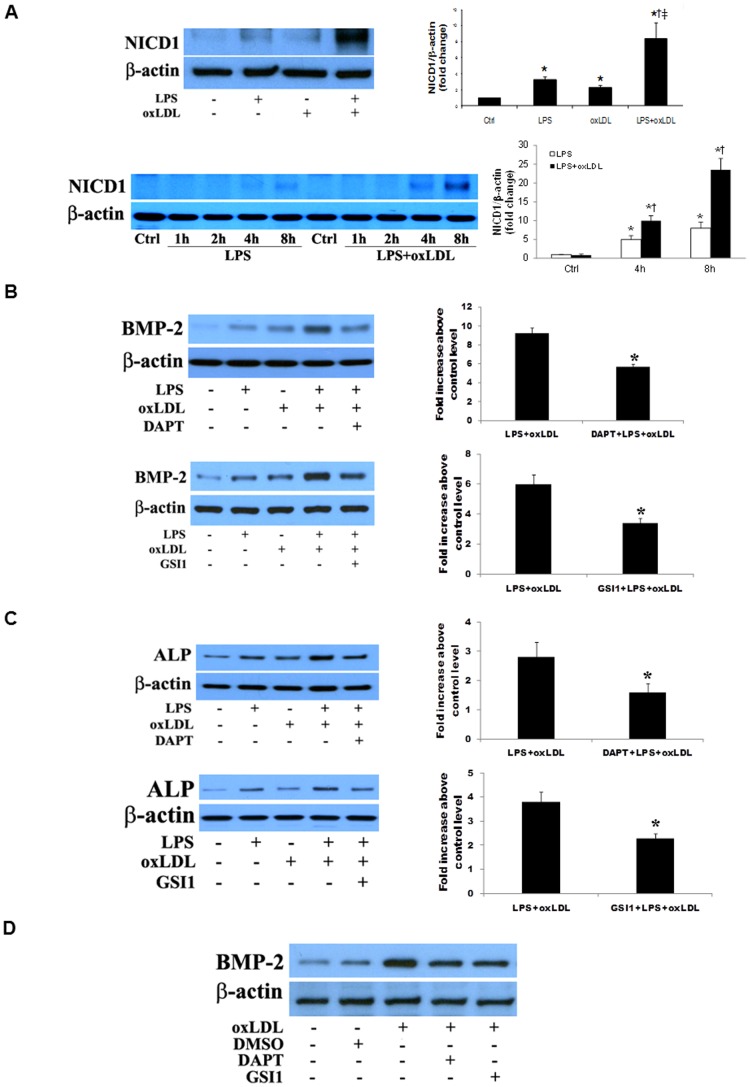
Notch1 is involved in the induction of the osteogenic responses by oxLDL and LPS plus oxLDL. **A**. Human AVICs were treated with LPS (0.10 µg/ml), oxLDL (20 µg/ml) or LPS plus oxLDL for 1–8 h. Representative immunoblots and densitometric data (n = 4) show increased NICD1 generation in cells treated for 8 h with LPS or oxLDL. Higher levels of NICD1 are detected in cells treated with LPS plus oxLDL. Time course data show that NICD1 is detectable at 4 h after exposing to LPS plus oxLDL and accumulates over time. *P<0.05 vs. untreated control; †*P*<0.05 vs. cells treated with LPS alone; ‡ *P*<0.05 vs. cells treated with oxLDL alone. **B and C**. Human AVICs were treated with LPS plus oxLDL in the presence or absence of γ-secretase inhibitor DAPT or GSI1. Representative immunoblots and densitometric data (n = 4) show that inhibition of Notch1 with DAPT or GSI1 reduces levels of BMP-2 (B) and ALP (C). *P<0.05 vs. cells treated with LPS plus oxLDL. **C**. Human AVICs were treated with oxLDL alone in the presence or absence of γ-secretase inhibitor DAPT or GSI1. Representative immunoblots of 3 separate experiments show that inhibition of Notch1 with DAPT or GSI1 reduces BMP-2 levels.

To determine the effect of oxLDL on Notch1 cleavage, we treated human AVICs with LPS alone, oxLDL alone and LPS plus oxLDL, and analyzed NICD1 levels. As shown in Figure 3A, NICD1 was detectable by immunoblotting after an exposure to either oxLDL alone or LPS alone for 8 h. Cells exposed to LPS plus oxLDL for the same period of time exhibited higher NICD1 levels. The results from time course experiments show higher NICD1 levels in cells treated with LPS plus oxLDL for 4 to 8 h (Figure 3A).

To determine the role of Notch1 in the augmented osteogenic responses to LPS plus oxLDL, we inhibited Notch1 cleavage using γ-secretase inhibitors, DAPT and GSI1. The protein levels of BMP-2 and ALP were decreased by 38.7–43.8% in cells treated with DAPT or GSI1 prior to stimulation with LPS plus oxLDL (Figures 3B and 3C). In addition, inhibition of Notch1 cleavage with DAPT or GSI1 also attenuated BMP-2 expression induced by oxLDL (Figure 3D).

**Figure 4:**
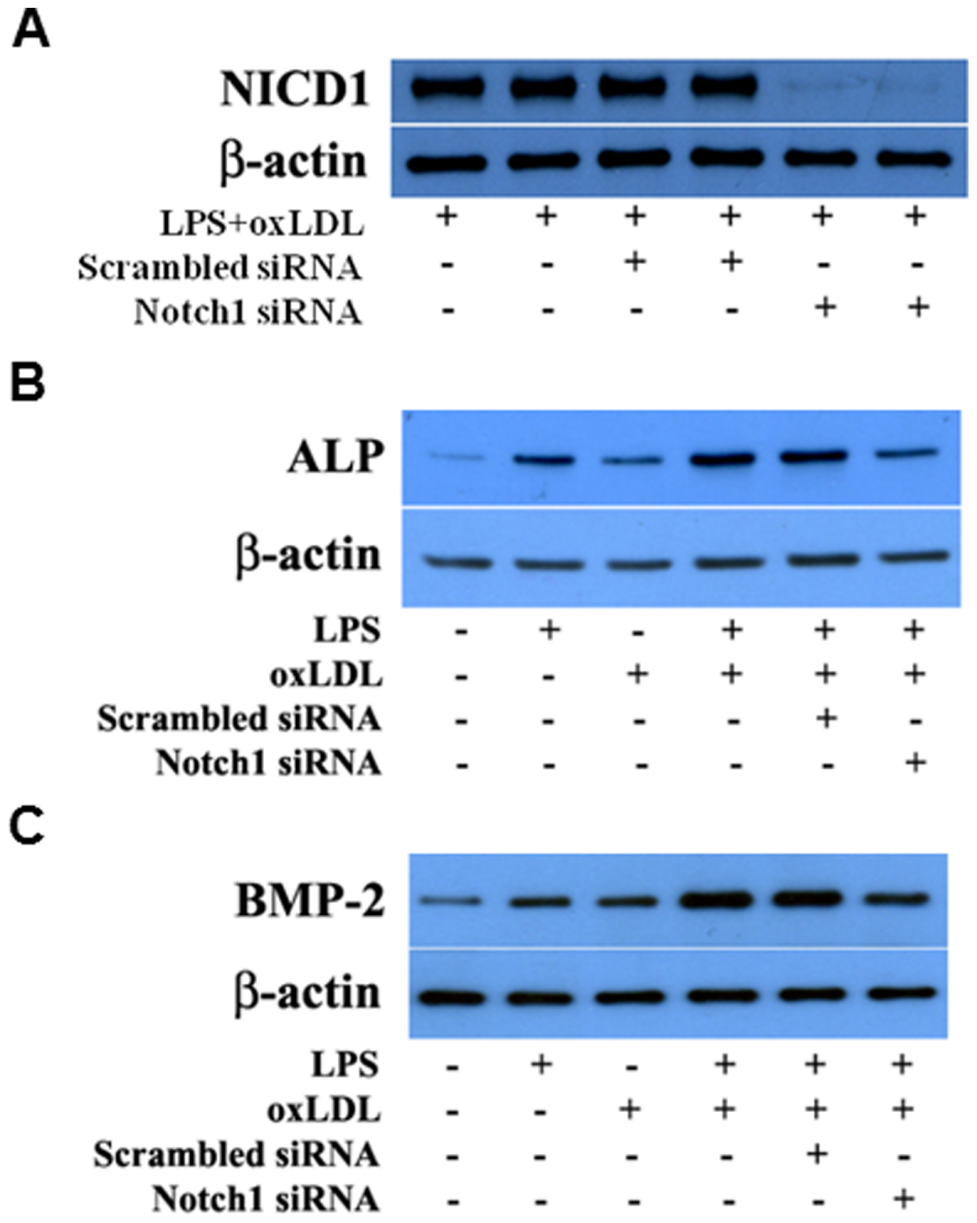
Notch1 knockdown reduces the expression of BMP-2 and ALP following stimulation with LPS plus oxLDL. Human AVICs were treated with Notch1-specific siRNA and then stimulated with LPS plus oxLDL. Representative immunoblots of 2 separate experiments show that knockdown of Notch1 results in decreased expression of BMP-2 and ALP following stimulation with LPS plus oxLDL that is associated with reduced levels of NICD1.

To further determine the role of Notch1 in the augmented osteogenic responses, we applied Notch1-specific siRNA to knockdown Notch1. Figure 4A shows that Notch1 knockdown markedly reduced the levels of NICD1 in cells treated with LPS plus oxLDL. Notch1 knockdown also attenuated the expression of BMP-2 and ALP following stimulation with LPS plus oxLDL (Figures 4B and 4C).

### OxLDL Enhances NF-κB Activation Though Modulation of Notch1 Activation

**Figure 5:**
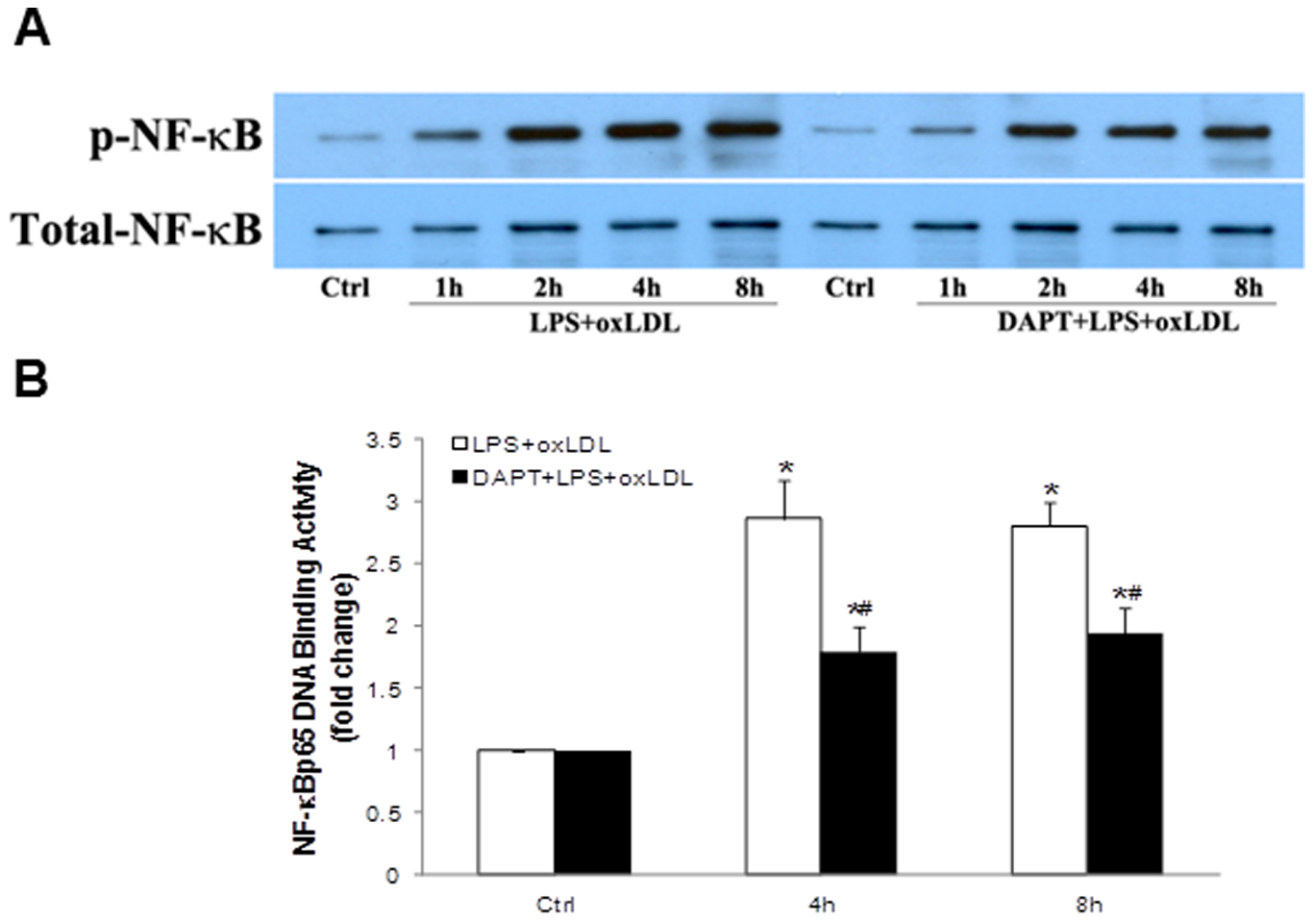
Inhibition of Notch1 cleavage attenuates NF-κB activation induced by LPS plus oxLDL. Human AVICs were treated with LPS plus oxLDL in the presence or absence of γ-secretase inhibitor DAPT. A representative immunoblot of 3 separate experiments and ELISA data (n = 4 in each group) show that NF-κB phosphorylation and DNA-binding activity are reduced when DAPT is present during stimulation with LPS plus oxLDL. *P<0.05 vs. untreated control; # *P*<0.05 vs. cells treated with LPS plus oxLDL.

To test whether Notch1 activation has a role in NF-κB activation, we added DAPT to cell cultured media 1 h prior to addition of oxLDL and LPS, and analyzed the levels of NF-κB phosphorylation and DNA-binding activity. Pre-treatment with γ-secretase inhibitor DAPT attenuated NF-κB p65 phosphorylation (Figure 5A), as well as NF-κB DNA-binding activity (Figure 5B), at all time points following treatment with LPS plus oxLDL. The results demonstrate a role of Notch1 in mediating the modulation of NF-κB activation by oxLDL.

### Inhibition of NF-κB Attenuates the Osteogenic Responses Induced by LPS plus Notch1 Ligand

**Figure 6:**
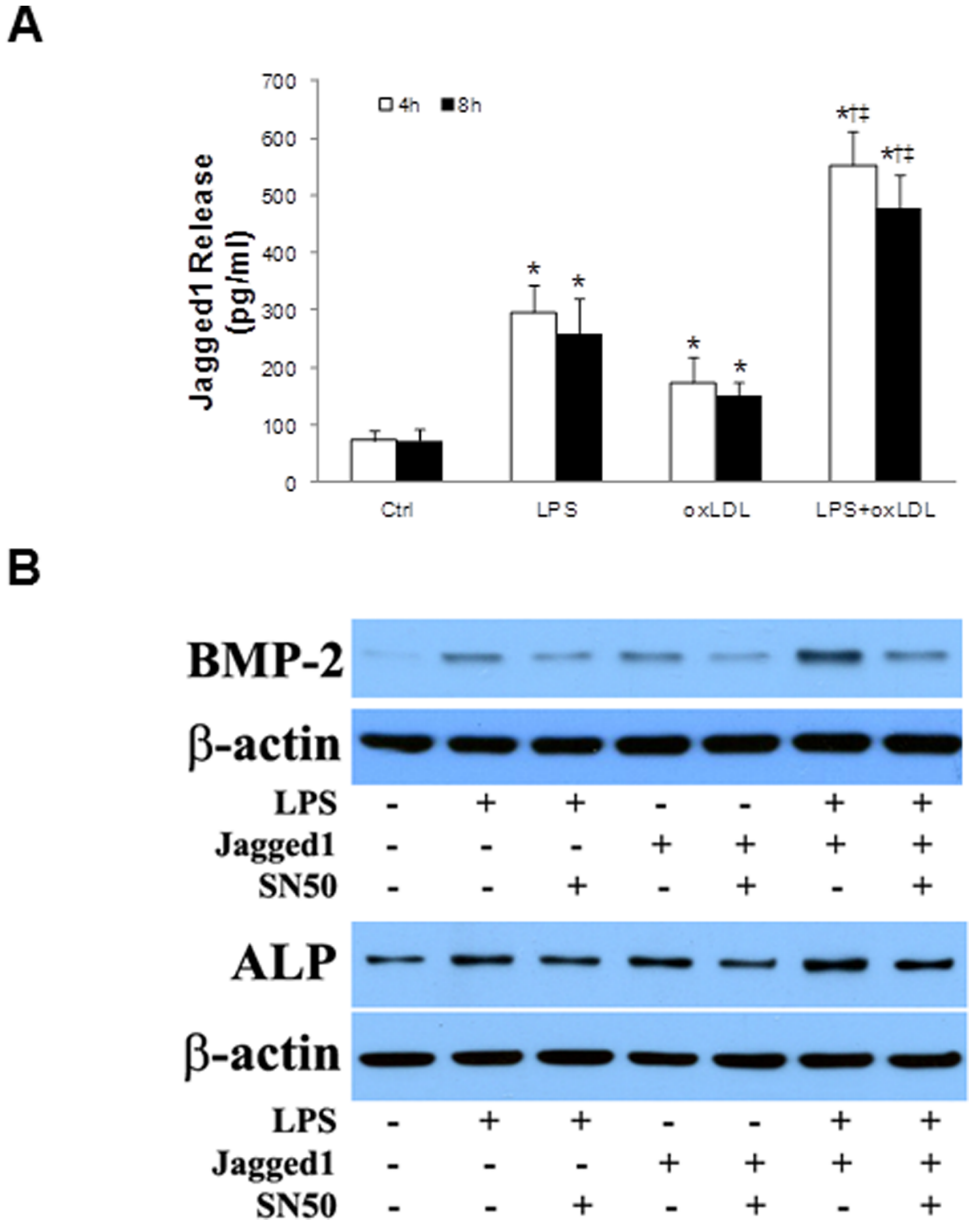
Inhibition of NF-κB suppresses the osteogenic responses induced by Jagged1 plus LPS. **A**. AVICs were treated with LPS (0.10 µg/ml), oxLDL (20 µg/ml) or LPS plus oxLDL for 4 or 8 h. ELISA data (n = 4 in each group) show that both LPS and oxLDL up-regulate Jagged1 secretion. Cells treated with LPS plus oxLDL release higher levels of Jagged1. *P<0.05 vs. untreated control; †*P*<0.05 vs. cells treated with LPS alone; ‡ *P*<0.05 vs. cells treated with oxLDL alone.

We previously reported that stimulation of TLR4 in human AVICs lead to the secretion of Notch1 ligand Jagged1 [26]. In the present study we examined whether oxLDL induces secretion of Jagged1 and whether stimulation of cells with LPS plus oxLDL causes exaggerated Jagged1 secretion. The results in Figure 6A show that cells exposed to LPS plus oxLDL released a greater amount of Jagged1 at 4 and 8 h while cells exposed to oxLDL alone exhibited a moderate release of Jagged1.

We then examined whether Jagged1 has a similar effect as oxLDL in augmenting the osteogenic responses. As shown in Figure 6B, levels of BMP-2 and ALP were higher in cells treated with LPS plus Jagged1 than those in cells treated with LPS alone or Jagged1 alone. Inhibition of NF-κB with the specific inhibitor SN50 attenuated not only the effect of LPS plus Jagged1, but also the effect of Jagged1 alone, indicating that Notch1 modulates the osteogenic responses in human AVICs in a NF-κB-dependent fashion.

## Discussion

Hyperlipidemia is recognized as a risk factor for CAVD [2], [32], [33]. Although CAVD is not commonly accompanied by atherosclerosis, oxLDL accumulation in calcific, stenotic areas of diseased aortic valves have been reported by a number of studies [13], [14], [15], [16]. Currently, the role of oxLDL in the pathogenesis and progression of CAVD remains unclear. The results of the present study demonstrate that oxLDL is pro-osteogenic to human AVICs and synergizes with LPS to induce augmented osteogenic responses. OxLDL augments the osteogenic responses through modulation of Notch1 and NF-κB activation. The pro-osteogenic effect of oxLDL and its interaction with a TLR4 agonist indicate that oxLDL plays a role in aortic valve calcification associated with CAVD.

### OxLDL is Pro-osteogenic to Human AVICs and Synergizes with TLR4 Agonist to Augment the Osteogenic Responses

Our previous study found that oxLDL up-regulates BMP-2 expression in human coronary artery endothelial cells through a mechanism involving NF-κB activation mediated by TLR2 and TLR4, although oxLDL does not induce cytokine production [17]. To understand whether oxLDL has a pro-osteogenic effect on human AVICs, we treated cells with oxLDL and examined the expression of BMP-2 and ALP. We observed that oxLDL up-regulates the expression of pro-osteogenic protein BMP-2. This finding is consistent with that obtained in human coronary artery endothelial cells and indicates that oxLDL is pro-osteogenic to human AVICs although oxLDL alone has a minimal effect on cellular ALP levels.

Several studies reported the presence of bacterial agents in diseased aortic valves [6], [7], [8], [9]. Moreover, human AVICs are found to exhibit osteogenic responses to pathogen-associated molecular patterns and danger-associated molecular patterns [4], [5], [27], [29]. To understand the effect of oxLDL on human AVIC osteogenic responses to pathogen-associated molecular patterns, we stimulated cells with LPS in the presence and absence of oxLDL. We observed that cells treated with LPS plus oxLDL produce markedly higher levels of BMP-2 and ALP in comparison to cells treated with LPS alone. The augmented osteogenic responses appear to be due to a synergistic effect of oxLDL and LPS since the increases in BMP-2 and ALP in cells treated with LPS plus oxLDL are much greater than the added increases in cells treated with LPS alone and in cells treated with oxLDL alone. Thus, the results show that oxLDL also augments the expression of pro-osteogenic proteins in AVICs exposed to TLR4 agonist LPS. Together, our results demonstrate that oxLDL is pro-osteogenic to human AVICs and synergizes with TLR4 agonist to augment the osteogenic responses. These findings in human AVICs indicate that oxLDL accumulation in aortic valves may contribute to the mechanism underlying the disease progression in CAVD.

Several studies have determined the effect of oxLDL priming on TLR4-mediated inflammatory response in monocytes and macrophages. Pre-treatment of monocytes or macrophages with oxLDL for 20 h or longer has been shown to enhance or suppress cellular inflammatory responses to subsequent TLR4 stimulation [18], [34], [35], [36]. The enhancement of LPS response is attributed to up-regulation of the expression of TLR4 and CD14 by oxLDL pre-treatment [18]. Cell priming is unlikely to contribute to the effect of oxLDL on the osteogenic responses in human AVICs observed in the present study since oxLDL is added 2 h prior to addition of LPS. This time interval is not sufficient to up-regulate TLR4 expression. We have reported that both TLR2 and TLR4 are involved in the up-regulation of BMP-2 expression by oxLDL in human coronary artery endothelial cells [17]. Several studies have demonstrated that TLR2 and TLR4 agonists synergistically up-regulate the expression of proinflammatory cytokines, such as TNF-α[37], [38]. It is likely that oxLDL synergizes with LPS to induce the osteogenic response by functioning as a TLR2 agonist. Alternatively, oxLDL may modulate the distribution of TLR4 on the cell surfaces. Further studies are needed to investigate these potential mechanisms of synergy. Nevertheless, we found the synergistic effect of oxLDL and LPS involve augmented intracellular signaling mechanisms.

### OxLDL Modulates NF-κB and Notch1 Activation to Augment the Osteogenic Responses to TLR4 Stimulation

In order to understand the mechanism by which oxLDL modulates the osteogenic responses in human AVICs, we determined the effect of oxLDL on activation of NF-κB and Notch1 since these pathways are involved in the regulation of the osteogenic responses in human AVICs [27]. The results of the present study show that oxLDL is capable of activating NF-κB and Notch1. In addition, oxLDL exaggerates NF-κB and Notch1 activation following TLR4 stimulation.

We have reported that NF-κB plays an important role in mediating oxLDL-induced BMP-2 expression in human coronary artery endothelial cells [17]. In the present study, we found that inhibition of NF-κB with either of the two inhibitors results in greater than 50% reductions in the expression of BMP-2 and ALP in human AVICs exposed to oxLDL and LPS. Thus, NF-κB plays a major role in mediating the osteogenic responses induced by LPS plus oxLDL in human AVICs.

Notch1 plays a role in vascular calcification [39]. Recent studies have linked TLR4 to Notch1 activation. In this regard, inhibition of γ-secretase, which processes Notch1 to release NICD1, reduces LPS-induced expression of pro-inflammatory cytokines in macrophages [24], [25]. We observed in the present study that inhibition of γ-secretase with either DAPT or GSI1 attenuates the expression of BMP-2 and ALP in cells exposed to LPS plus oxLDL. Similarly, Notch1 knockdown reduces the levels of BMP-2 and ALP in cells treated with LPS plus oxLDL. Since oxLDL induces the generation of NICD1, it is likely that enhancement of Notch1 activation is a mechanism by which oxLDL augments the osteogenic responses in human AVICs. Indeed, the results from the experiments using LPS and Notch1 ligand Jagged1 show that Jagged1 also has a synergistic effect with LPS in the induction of the expression of BMP-2 and ALP in human AVICs.

### OxLDL Modulates Notch1 Activity to Augment NF-κB-dependent Osteogenic Responses in Human AVICs

Previous studies found that Notch1 modulates NF-κB activity in macrophages and cancer cells [24], [40]. We recently found in human AVICs that Jagged1 enhances NF-κB activation, and NICD interacts with IKK [26]. The results of the present study show that inhibition of Notch1 attenuates NF-κB phosphorylation and DNA-binding activity in cells exposed to oxLDL and LPS. In addition, inhibition of NF-κB attenuates the osteogenic responses induced by Jagged1 and Jagged1 plus LPS. These results indicate that NF-κB is a downstream target of activated Notch1. This is consistent with previous studies in cancer cells where a Notch1 mutation that leads to constitutive generation of NICD1 results in persistent NF-κB activation [41]. Thus, the Notch1-NF-κB axis plays a role in the augmentation of the osteogenic responses by oxLDL.

It seems that a mechanism that contributes to the enhancement of Notch1 activation by oxLDL is the up-regulated secretion of Notch1 ligands. Indeed, we observed that cells exposed to LPS plus oxLDL secrete higher levels of Jagged1 than cells exposed to oxLDL alone or LPS alone. Since oxLDL can interacts with TLR2 and TLR4 [17], the mechanism by which oxLDL enhances Notch1 activation may also involve its direct interaction with these two innate immunoreceptors. Further studies are needed to investigate the molecular mechanism underlying the modulation of the Notch1-NF-κB pathway by oxLDL in AVICs.

Taken together, the results of the present study demonstrate that oxLDL induces the expression of pro-osteogenic protein BMP-2 in human AVICs. Further, oxLDL synergizes with TLR4 agonist LPS to exaggerate human AVIC production of BMP-2 and ALP. The augmented production of osteogenic mediators involves enhanced activation of Notch1 and NF-κB, and the influence of oxLDL on the Notch1-NF-κB cascade appears to play a role in the augmentation of the osteogenic responses. These novel findings demonstrate that oxLDL modulates AVIC osteogenic responses to pro-inflammatory stimulation and indicate a potential role of oxLDL in the multifactorial mechanism underlying CAVD pathogenesis and progression.
